# Improved surgical exposure and early clinical outcomes using a femoral-release-first technique in direct anterior approach during total hip arthroplasty

**DOI:** 10.1186/s13018-023-04334-y

**Published:** 2023-11-18

**Authors:** Hua-zhang Xiong, Li-dan Yang, Gang Bao, Jia-chen Peng, Zhi-hong Liu

**Affiliations:** 1 Department of Orthopedic Surgery, Affiliated Hospital of Zunyi Medical University, 149# Dalian Road, Zunyi, 563003 People’s Republic of China; 2 Department of Orthopedic Surgery, People’s Hospital of Yinjiang Tujia and Miao Autonomous County, 52# Xiyuan Road, Yinjiang, 555200 People’s Republic of China; 3 Department of Orthopedics, Ruijin Hospital, Shanghai Jiao Tong University School of Medicine, 197# Ruijin Second Road, Shanghai, 200025 People’s Republic of China

**Keywords:** Direct anterior approach, Total hip arthroplasty, Comparison, Traditional approach, Femoral-release-first

## Abstract

**Background:**

Total hip arthroplasty (THA) performed using the direct anterior approach (DAA) has demonstrated favourable early-, mid-, and long-term outcomes. However, the traditional femoral release technique remains technically demanding and is associated with challenges and a heightened risk of complications. This study aimed to compare the clinical outcomes of patients who underwent THA with DAA performed using either the femoral-release-first (FRF) or the traditional approach (TA) strategy.

**Methods:**

A retrospective analysis of demographics, clinical and radiological outcomes, and occurrence of complications was performed using data from 106 patients between 2018 and 2019. The patients were categorised into two groups: FRF (44 hips) and TA (69 hips).

**Results:**

The FRF group showed a reduced operative time, haemoglobin (Hb) drop, postoperative hospital stay, and more optimal acetabular cup anteversion angles. Furthermore, during the first 2 months postoperatively, the FRF group demonstrated superior visual analogue scale, Harris Hip, and Oxford Hip scores. In the TA group, two hips experienced greater trochanter fractures, and one experienced delayed incision healing.

**Conclusions:**

Compared with the TA, employing the FRF strategy during THA with DAA resulted in improved outcomes within the first 2 months postoperatively and comparable functional recovery beyond this period. The FRF method exhibited advantages such as favourable acetabular exposure and alignment and a reduced risk of complications. Therefore, the FRF strategy may be a favourable option.

## Background

Total hip arthroplasty (THA) is the primary surgical intervention for terminal-stage osteoarthropathy and has demonstrated excellent clinical outcomes [1]. The choice of surgical approach for THA is a crucial factor influencing the therapeutic outcomes [2, 3]. An optimal operative approach should strive for minimal invasiveness, early functional recovery, and reduced complications, benefiting the patient and conserving medical resources [4]. Among various approaches, the direct anterior approach (DAA) has emerged as a promising method, offering less trauma and faster recovery than other approaches [3, 5, 6].

The traditional approach (TA) involving routine femoral release after cup installation has been extensively studied [7–9]. Patients who underwent THA with DAA using the TA demonstrated good early-, mid-, and long-term outcomes [1, 8, 10]. However, the traditional femoral release approach remains technically demanding and is associated with a higher risk of complications, including greater trochanter fractures and muscular impairment [2, 7]. Appropriate elevation and exposure of the proximal femur are required to perform the femoral broach and stem installation.

For patients with congenital developmental dysplasia of the hip, employing a surgical technique involving complete femoral release before cup installation, which is defined as “femoral-release-first (FRF)”, can facilitate acetabular cup installation and femoral release, lead to shorter operative time, and reduce complications. The FRF method involves complete femoral release before cup installation, creating an appropriate space between the acetabulum and the proximal femur, thereby enhancing surgical ease and precision. However, to our knowledge, no comparative studies have investigated the postoperative outcomes of the FRF method and TA in patients who underwent THA using DAA.

Therefore, this retrospective study aimed to assess the differences in clinical and radiological outcomes and complications between the FRF method and TA in patients who underwent THA using DAA. We hypothesised that the FRF method would provide better postoperative outcomes and that patients using the FRF method would recover faster.

## Methods

This study was approved by the ethics committee of the Affiliated Hospital of Zunyi Medical University (KLL-2022-613). All methods were performed in accordance with the Chinese Ethical Guidelines for Medical and Biological Research Involving Human Subjects, and each participant provided written informed consent. The surgical technique was extracted from the surgical notes for each patient. All surgeries in this study were performed using DAA by a senior surgeon at our institution to minimise the influence of learning-curve-related complications. The inclusion criteria were as follows: presence of end-stage hip osteoarthritis (OA); age of 20–80 years; body mass index (BMI) < 30 kg/m^2^ [11]; and American Society of Anesthesiologists (ASA) grade < 4. This study met the diagnostic criteria for hip OA provided by the Guidelines for the Diagnosis and Treatment of OA [12]. The exclusion criteria included history of single-stage bilateral surgery, hip surgery or traumas, lumbar spinal fusion, the presence of femoral head osteonecrosis, serious organic or infectious diseases, no satisfactory imaging data, loss to follow-up, a follow-up period of < 25 months, and the presence of other prostheses.

All surgical notes were carefully reviewed to identify the surgical technique used (TA or FRF). Individuals who underwent treatment between January 2018 and December 2019 were assessed for inclusion in this study. During the study period, the FRF method and TA were performed without a systematic process for assigning a surgical approach. Notably, the frequency of the FRF application increased significantly during this timeframe. The learning curves for both methods were completed in accordance with a previously defined learning curve [13].

All the included patients received the ACT cup-ML-TH stem system (AK, Beijing, China) for THA. Cementless press-fit components were uniformly applied to the acetabulum and femur. Using standardised prostheses allowed us to eliminate any additional procedures required for initial THA, facilitating the creation of comparable groups for analysis.

### Surgical technique

**Fig. 1:**
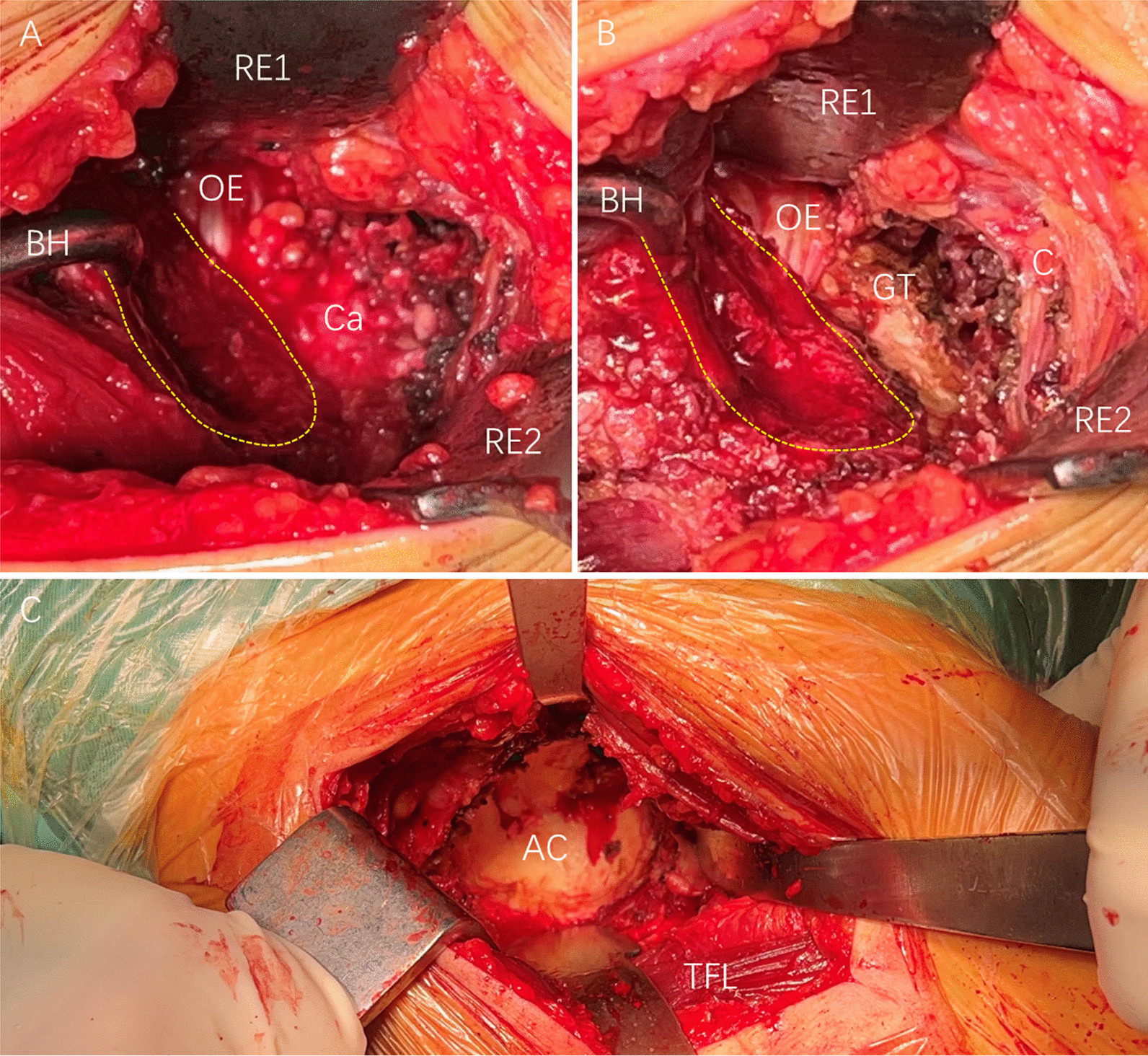
Intraoperative images showing the femoral neck (yellow dotted line) and obturator externus locations. The greater trochanter was placed at a position of external rotation, adduction, and extension of the hip joint before (**A**) or after (**B**) release. Intraoperative imaging shows good acetabular exposure (**C**). AC, acetabulum; BH, bone hook; Ca, capsule; GT, greater trochanter; OE, obturator externus; RE, retractor; TFL, tensor fasciae latae

**Fig. 2:**
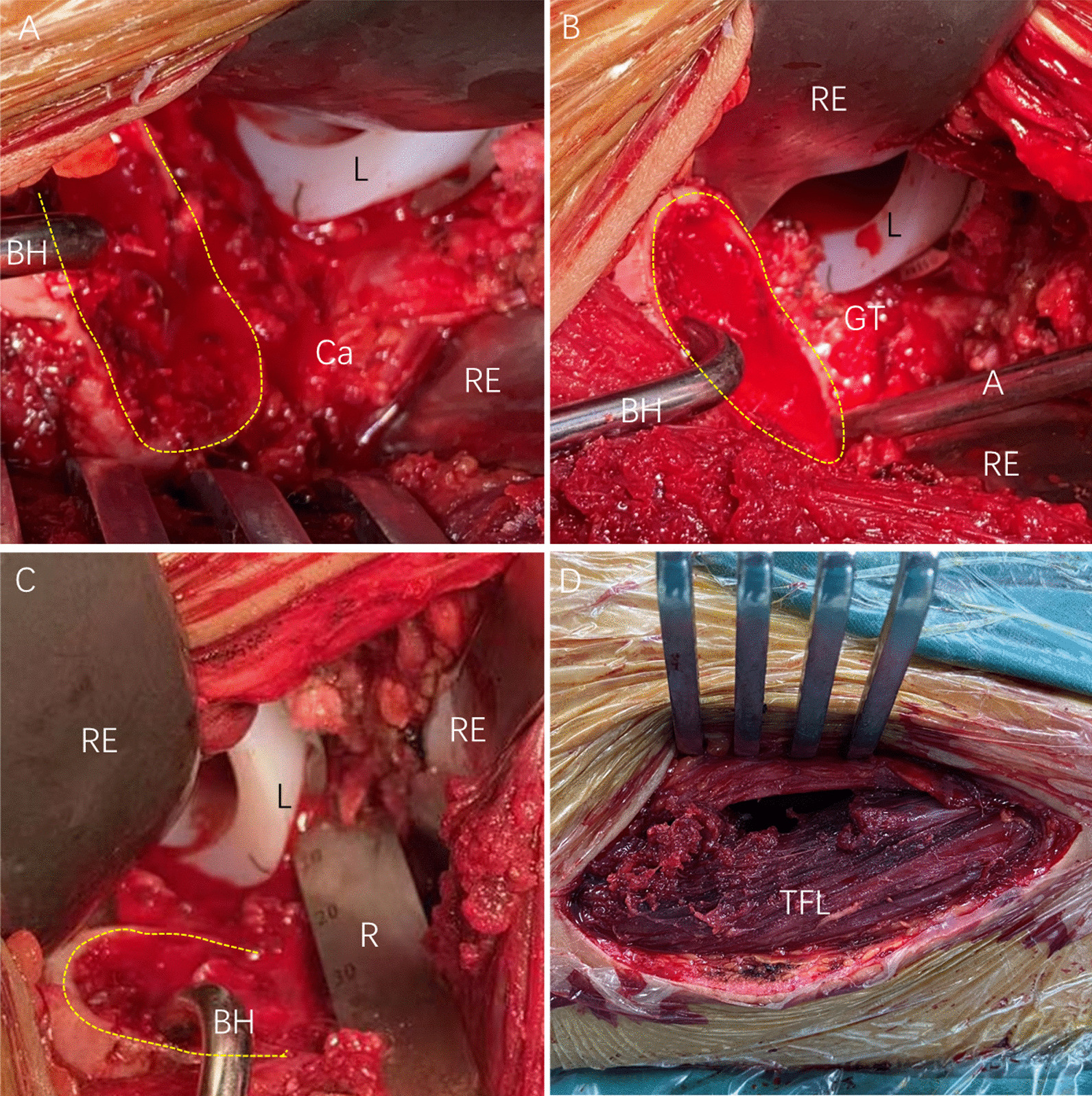
**A** Intraoperative image showing the location of the femoral neck (yellow dotted line) and the liner before release. **B** Intraoperative image showing the location of the greater trochanter and the liner after release at the greater trochanter. The posterior edge of the greater trochanter is located behind the liner (L) at a position of external rotation, adduction, and extension of the hip joint. **C** Intraoperative image showing the high edge of the liner, which decreases the space between the acetabulum and the femoral neck by 10 mm. **D** Intraoperative image showing an iatrogenic injury of the TFL. A, aspirator; BH, bone hook; Ca, capsule; GT, greater trochanter; L, liner; R, ruler; RE, retractor

All patients underwent general anaesthesia and had well-controlled blood pressure. All surgeries were performed with the patient in the supine position on an orthopaedic table under fluoroscopy. The affected hip was placed on the far ipsilateral side of the table, and a levelled pelvis was achieved. The symphysis pubis was placed at the flexion point of the operating table, which allowed the extension of the hip joint to improve the angle of insertion of the femoral component and instruments during the procedure. The flexion point was checked before the sterile preparation of the affected limb. The surgical procedure was performed as described by Post et al. [14] and Chughtai et al. [8]. An incision was made approximately 2 cm distal and 2 cm lateral to the anterior superior iliac spine, which extended in the direction of the fibular head and was approximately 8–10 cm in length. The fascia of the tensor fasciae latae (TFL) muscle was split in a line along the muscle fibres to allow blunt separation and access to the Hueter gap between the sartorius and TFL muscles. The ascending branch of the lateral femoral circumflex artery was carefully ligated, and the reflected head of the rectus was released to improve exposure. A capsulectomy was performed, the capsule dissection was initiated in line with the femoral neck, the inferior medial and superior lateral capsular femoral flaps were removed, and the capsule excision was considered the primary release. Subsequently, the lesser trochanter initiated the release of the pubofemoral ligament inferomedially. The femoral neck was cut, and the femoral head was removed. In the FRF group, after performing a femoral neck cut, the affected limb was first positioned for external rotation and adduction, and the table was flexed to allow hip extension. A Mueller retractor (RE1; Fig. 1A, B) was placed over the posterior aspect of the femoral neck to distract the medial tissues. Another Mueller retractor (RE2; Fig. 1A, B) was positioned over the superior site of the greater trochanter with the hip abductor muscles behind it to allow separation of the muscle and joint capsule and to improve the appropriate exposure of the proximal femur. A bone hook (BH; Fig. 1A, B) was placed in the femoral canal to distract the proximal femur towards the anterolateral aspect of the incision. The residual pubofemoral ligament, posterior capsule, superior capsule, and conjoined tendon were gradually released in order, as required. During the procedure, the surgeon intermittently discontinued the release to determine the height at which the proximal femur could be elevated. Once the mouth of the femoral neck was above the anterior rim of the acetabulum, which permitted sufficient exposure for broaching and stem installation (Fig. 1B), the femoral release was completed. The release of the abductor musculature, piriformis, and obturator externus tendons was avoided. Subsequently, the acetabular cup procedure was performed, and offset acetabular reamers and cup insertion handles were used to improve the reaming and cup installation. Furthermore, femoral procedures were performed. In the TA group, the necessary femoral release was completed after the acetabular procedure was performed. The acetabular and femoral procedures were as previously described [14], as shown in Fig. 2. Finally, the sizes and positions of the femoral stem and acetabular cup were confirmed radiographically. The acetabular cup was placed in a safe zone of 30°–50° of inclination and 5°–25° of anteversion [15]. The incision was sutured layer by layer. No drainage tubes were used.

### Perioperative management

All patients received pain management, prophylactic antibiotics, and postoperative prophylactic antithrombotic management. All patients were allowed in terms of hip mobility, and ambulation was initiated 1 day postoperatively. The goal of discharge to home was set for postoperative Day 2. Haemoglobin (Hb) levels were measured within 24 h postoperatively. A blood transfusion was required when the Hb level was < 70 g/L. Follow-up was performed according to our institution’s standard postoperative schedule at 1, 2, 3, 6, and 12 months and annually afterwards.

### Data collection

#### Demographic and clinical characteristics

Patients' data were collected, including sex, age, BMI, ASA grade, involved side, Hb drop, blood transfusion, operative time, postoperative hospital stay, and follow-up time. The operative time was measured from the initiation of the skin incision to the completion of the incision suturing. The Hb drop was calculated using the preoperative Hb value minus the value on postoperative Day 1.

### Clinical outcomes

Clinical outcomes were evaluated using the Harris Hip Score (HHS) [16], Oxford Hip Score (OHS) [17], and visual analogue scale (VAS) score [18]. The HHS was used to assess hip function recovery, with scores ranging from 0 (worst) to 100 points (best). The OHS was used to evaluate hip pain and function, with scores ranging from 0 (worst) to 48 (best). The VAS score was used to assess pain on a scale of 0–10 (0 = no pain and 10 = worst pain). The postoperative patient-reported outcomes were recorded and analysed to compare the differences between the two surgical strategies.

### Radiographic evaluations

All patients’ preoperative, 1-day postoperative, and 3-month postoperative anteroposterior hip radiographs were routinely obtained using a standardised technique [19]. A position with a 20° internal rotation of the hip joint was applied to achieve a standardised and reproducible image during follow-up. The X-ray tube was placed perpendicularly at a 1-m distance from the table. Radiographs obtained 3 months postoperatively were used to evaluate stem alignment (graded as varus, neutral, or valgus) and cup alignment (inclination and anteversion angles) [20, 21]. The inter-teardrop line was used as the reference line for measurement of the acetabular cup inclination angle, and a deviation > 3° from the axis of the femur was defined as a valgus or varus position [22]. The periprosthetic radiolucent lines and osteolysis were evaluated in the femur according to the 14 zones of Gruen [23] and in the acetabulum according to DeLee and Charnley [24, 25]. Subsidence of the femoral stem was defined as any change in distance between the stem shoulder and the tip of the greater trochanter on the final follow-up radiographs compared with immediate postoperative radiographs [26]. Femoral stem loosening was defined as subsidence > 5 mm [27], progressive femoral stem tilt [28], radiolucent lines > 2 mm at the bone-stem interface [23], or multiple bone cavitations [28, 29]. Acetabular cup loosening was defined as a tilt > 5° or radiolucent lines > 2 mm at the bone-component interface in two or three DeLee and Charnley zones on the final follow-up radiographs compared with the immediate postoperative radiographs [23, 24]. All radiographs were evaluated by two independent radiologists blinded to the clinical treatment and outcomes. Inter-observer reliability between the two radiologists was evaluated using interclass correlation coefficients (ICCs) interpreted as follows: > 0.9: excellent; 0.75–0.9: good; 0.50–0.74: fair; and < 0.50: poor. ICCs were interpreted using previously reported semi-quantitative criteria [30].

### Perioperative complications

Data on complications, including greater trochanter fracture, lateral femoral cutaneous nerve injury, incision-related conditions (oozing, delayed healing, and infection), dislocation, and venous thromboembolism (VTE), were collected.

### Statistical analyses

The sample size for the present study was calculated using Slovin’s formula, as previously described by Ellen [31]. The participants’ size was determined according to the number of patients with hip OA reported by Li et al. [32]; *N* was 60 patients for 6 months, and *e* was 0.05 at a 95% confidence interval; 60 patients were required for the present study. Each group consisted of at least 30 patients. Statistical tests were performed using SPSS® software version 22 (SPSS Inc., Chicago, IL, USA) by a researcher blinded to surgical procedures and data collection. All values were expressed as means with standard deviations. The Kolmogorov–Smirnov test was performed on each continuous variable to determine normality. The Mann–Whitney U test was used for continuous variables. Differences between sets of categorical data were analysed using Fisher’s exact probability or Pearson's Chi-square test for outcomes between the FRF and TA groups. Statistical significance was set at *p* < 0.05.

## Results

### Patients

**Fig. 3:**
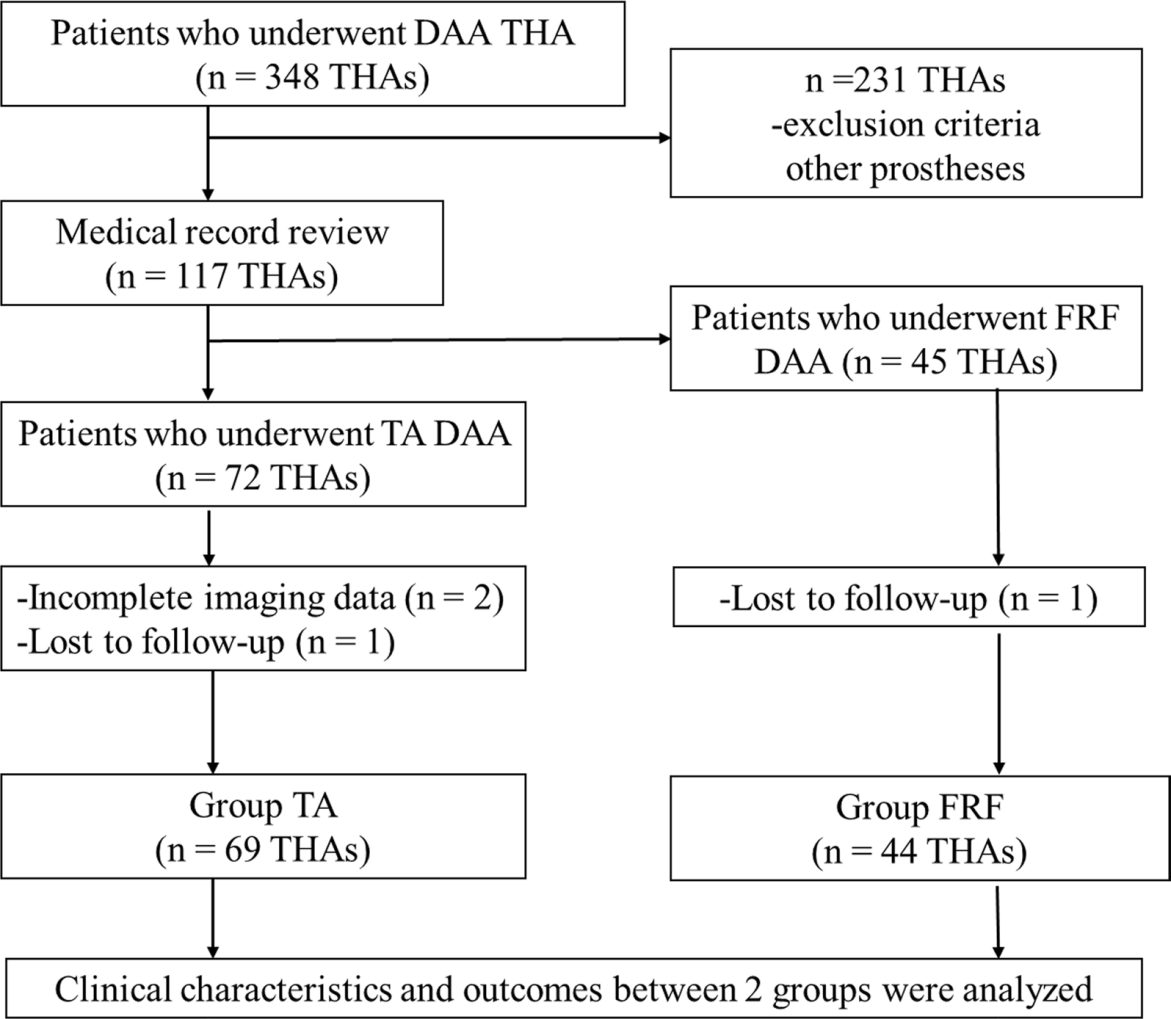
Flowchart of patient enrolment. DAA, direct anterior approach; FRF, femoral-release-first; TA, traditional approach; THA, total hip arthroplasty

Of the initial 348 screened cases, 235 were excluded based on the exclusion criteria **(**Fig. 3**)**. Subsequently, a retrospective review of 113 THAs (106 patients who underwent THA with DAA) between January 2018 and December 2019 was conducted. Each THA required a minimum follow-up period of 25 months (mean: 36.0 ± 6.5 months, range: 25–48 months) for inclusion in this study.

Of the 113 THAs, 44 were performed using the FRF method, whereas 69 were performed using the TA. Demographic data, including age, sex, BMI, ASA grade, and affected side, showed no significant differences between the two groups. Additionally, the Hb drop, operative time, postoperative hospital stay, and total follow-up time showed significant differences between both groups (*p* < 0.05) (Table 1).

**Table 1:** Demographics and characteristics of patients^ψ^

Characteristic	FRF group (*N* = 44)	TA group (*N* = 69)	*p* value
Age (y)	56.1 ± 12.8	55.3 ± 10.6	0.722^a^
Sex (M/F)	30/14	39/30	0.215
Body mass index (kg/m^2^)	24.1 ± 2.9	25.0 ± 2.8	0.075^a^
ASA grade (I/II/III)	25/10/9	40/13/16	0.862^b^
Affected side (right/left)	20/24	36/33	0.585^b^
**Operative time (Min)**	**60.8 ± 4.6**	**68.8 ± 5.4**	**0.000**^**a**^
**Postoperative hospital stay (Day)**	**2.8 ± 0.8**	**3.3 ± 1.7**	**0.003**^**a**^
**Follow-up time (Month)**	**29.4 ± 3.3**	**40.3 ± 3.9**	**0.000**^**a**^
Blood transfusion	3 (6.8%)	5 (7.2%)	1.000^b^
**Hb drop (g/L)**	**32.4 ± 6.7**	**35.2 ± 6.7**	**0.003**^**a**^

^ψ^Continuous variables are expressed as the mean and standard deviation. Categorical variables are presented as numbers with percentages in parentheses

^a^Independent‑sample Mann–Whitney U test

^b^Pearson's Chi-square test. The boldface indicates statistical significance. ASA, American Society of Anesthesiology; FRF, femoral-release-first; Hb, haemoglobin; Min, minutes; TA, traditional approach

### Clinical outcomes

The FRF group demonstrated better postoperative outcomes regarding HHS, OHS, and VAS scores than the TA group. The differences between the FRF and TA groups were statistically significant (*p* < 0.05) during the first 2 months postoperatively; however, no significant differences were observed afterwards (Table 2**)**.

**Table 2:** Postoperative functional recovery was measured in patients who underwent FRF or TA DAA for THA^ψ^

Outcome	FRF group (*N* = 44)	TA group (*N* = 69)	*p* value
VAS			
Preoperative	6.9 ± 0.9	7.0 ± 0.9	0.317
Postoperative			
**1 month**	**2.3 ± 0.8**	**2.8 ± 0.8**	**0.003**^**a**^
**2 months**	**1.4 ± 0.8**	**1.8 ± 0.8**	**0.004**^**a**^
3 months	0.75 ± 0.69	0.80 ± 0.70	0.726^a^
Last follow-up	1.0 ± 0.8	1.0 ± 0.7	0.793
HHS			
Preoperative	56.1 ± 2.7	55.8 ± 3.4	0.891
Postoperative			
**1 month**	**79.0 ± 4.7**	**75.0 ± 3.5**	**0.001**^**a**^
**2 months**	**83.5 ± 7.5**	**78.0 ± 4.5**	**0.002**^**a**^
3 months	89.6 ± 4.9	88.2 ± 4.4	0.100^a^
Last follow-up	93.8 ± 2.7	93.1 ± 4.4	0.297
OHS			
Preoperative	19.9 ± 2.6	20.7 ± 3.0	0.197
Postoperative			
**1 month**	**33.6 ± 2.3**	**32.8 ± 2.0**	**0.037**^**a**^
**2 months**	**37.4 ± 1.8**	**36.0 ± 1.5**	**0.000**^**a**^
3 months	42.1 ± 2.0	41.6 ± 2.8	0.199^a^
Last follow-up	42.6 + 1.1	42.3 ± 1.4	0.190

^ψ^ Continuous variables are expressed as the mean and standard deviation. ^a^ Independent‑sample Mann–Whitney U test. The boldface indicates statistical significance. DAA, direct anterior approach; FRF, femoral-release-first; HHS, Harris Hip Score; OHS, Oxford Hip Score; TA, traditional approach; THA, total hip arthroplasty; VAS, visual analogue scale

### Radiological outcomes

The FRF group showed better anteversion alignment of the acetabular cup than the TA group, and the difference was statistically significant (*p* < 0.05) (Table 3) based on the 3-month radiographs. Notably, no significant differences in cup inclination were observed between the two groups, and no signs of cup migration were observed in either group. Similarly, there were no significant differences in the varus, neutral, or valgus alignments of the femoral stem between the FRF and TA groups (Table 3). No radiological evidence of femoral stem loosening was observed in either group. The ICCs for anteversion of the acetabular cup, inclination of the acetabular cup, alignment (varus, neutral, and valgus) of the femoral stem, and subsidence were 0.938, 0.976, 1.0, and 0.955, respectively.

**Table 3:** Postoperative radiological measurements and complications between the two groups^ψ^

Measurements	FRF group (*N* = 44)	TA group (*N* = 69)	*p* value
Acetabular cup			
Inclination angle (°)	41.3 ± 2.2	42.0 ± 2.6	0.105^a^
**Anteversion angle (°)**	**15.2 ± 1.6**	**19.8 ± 3.9**	**0.000**^**a**^
Femoral stem			
Varus/neutral/valgus	3/41/0	5/64/0	1.000^b^
Subsidence (mm)	0.89 ± 0.62	0.93 ± 0.63	0.733^a^
**Complications**			**0.000**^**b**^
Greater trochanter fracture	0 (0%)	2 (2.9%)	
Delayed incision healing	0 (0%)	1 (1.4%)	

^ψ^ Continuous variables are expressed as the mean and standard deviation. Categorical variables are presented as numbers with percentages in parentheses

^a^Independent‑sample Mann–Whitney U test

^b^Pearson's Chi-square test. The boldface indicates statistical significance. FRF, femoral-release-first; TA, traditional approach

### Perioperative complications

Complications were observed in the TA group, including two greater trochanteric fractures and one delayed wound healing (Table 3). No other postoperative complications, such as infection or VTE, were observed.

## Discussion

The present study showed that the FRF strategy has notable advantages over the TA, including shorter operative time, decreased blood loss, a shorter postoperative hospital stay, and optimal cup anteversion with a better VAS score, HHS, and OHS than the TA in the first 2 months.

These outcomes showed significant differences, particularly the VAS score, HHS, and OHS, indicating that the FRF method for DAA THA achieved better early recovery than the TA. This suggests that surgery-induced muscle injury or inflammation may have occurred less in the FRF group. The advantages of the FRF method for DAA THA also include a lower Hb drop and fewer perioperative complications.

Operative time is an important factor that can affect clinical outcomes. Notably, several studies have reported that a longer operative time is associated with increased blood loss [33], whereas a shorter operative time is associated with reduced length of hospital stay and risk of readmission [34, 35]. In the present study, the TA group had a longer operative time and postoperative hospital stay, a higher Hb drop, and delayed incision healing. This is consistent with the findings of previous studies [4, 33–35]. Worse outcomes, possibly due to the femoral side procedure, were associated with the smaller space between the acetabular liner and femoral neck after cup placement when the proximal femoral release was performed in the TA group. The greater trochanter was blocked by the liner, which affected the elevation of the proximal femur when the femoral release was performed. The space further decreased at 90° external rotation of the femur because the greater trochanter was more posterior than the femoral neck. The smaller space makes the femoral release procedure more difficult, inducing the need for more time for femoral release and the occurrence of more complications (blood loss and long-term stretch injury to muscles). Furthermore, excessive release may occur because the blocked greater trochanter affects the elevation of the proximal femur, which may be mistaken for an inadequate release. The FRF method could also contribute to the shortened operative time in the FRF group. Furthermore, fewer surgical injuries and better clinical outcomes (VAS score, HHS, and OHS) were observed in the FRF group in the first 2 months.

The present study showed that the FRF method for DAA THA achieved optimal acetabular cup alignment. The TA group showed a higher anteversion of the acetabular cup, which was approximately 4.6° larger than that in the FRF group. In the FRF group, the femoral release was not only easily performed because there was no block of the acetabular liner but also because it made the acetabular procedure easier. The release aimed to elevate the proximal femur during the femoral procedure. Similarly, the femur can be further pushed posteriorly after release, increasing the space during acetabular procedures (Fig. 1C), allowing access to the acetabular instruments, and allowing easier installation of the acetabular cup. When installing the cup, its handle was more likely to be pushed posteriorly, as reported for the femur. Previous studies have reported that cup anteversion has a crucial effect on THA [36–38], which might be confirmed by the better postoperative VAS score, HHS, and OHS within the first 2 months in the FRF group. This indicates that the FRF method for DAA THA allows for accurate installation of the acetabular cup because of better acetabular exposure.

In the present study, a higher risk of intraoperative greater trochanter fracture was observed in the TA group (two cases) than in the FRF group (zero cases), possibly due to the forced elevation of the proximal femur with incomplete release during the TA [7, 39]. Rueckl et al. [7] reported that routine release of the conjoint tendon during the DAA for THA was associated with a lower risk of greater trochanter fracture than “release-on-demand” for the external rotator tendon. Knoth et al. [39] speculated that this higher rate of greater trochanter fracture resulted from the larger lever arm required to expose and elevate the proximal femur to insert the femoral stem and broaching instruments. Consistent with previous studies, the present study suggests that incomplete femoral release and forced elevation of the proximal femur are risk factors for greater trochanteric fractures. These findings suggest that the FRF method for DAA can reduce the risk of greater trochanteric fractures than the TA. These findings prove our hypothesis that the FRF method would provide better postoperative results in the first 2 months with fewer complications and that patients who underwent the FRF method would recover faster.

Notably, several studies on patients who underwent THA reported a significant annual increase in healthcare costs postoperatively because of the ageing population [40, 41]. Subsequently, Enhanced Recovery After Surgery (ERAS) was introduced to the management of patients who underwent THA to ensure reduced postoperative length of hospital stay, shorter convalescence, and rapid functional recovery without increasing morbidity and mortality [42, 43]. Ripolles-Melchor et al. [44] reported that an effective pathway for ERAS for THA consists of regional or local analgesia, anaemia and bleeding management, and early mobilisation as the basis of the care trajectory. In the present study, the FRF group had a lower Hb drop (anaemia and bleeding management), less pain (regional or local analgesia), better functional outcomes (early mobilisation), and fewer complications (morbidity), consistent with the results of previous studies. In addition, the shorter operative time and postoperative hospital stay increase the efficiency of the operative theatre and ward to reduce medical costs, and a shorter operation theatre stay and incision exposure further decrease the risk of infection and deep venous thrombosis [4]. Therefore, the FRF method used in the present study plays an important role in promoting ERAS and reducing medical costs.

Furthermore, the DAA for THA using the intermuscular planes has been associated with less perioperative pain, less muscle damage, rapid recovery, and a lower risk of early revision and dislocation [45, 46]. Despite the advantages of DAA, comparative studies evaluating the postoperative outcomes between the FRF method and TA for DAA are lacking. The present study addresses this knowledge gap, and we found similar primary clinical results between the FRF method and TA for DAA after the first 2 months postoperatively, including radiological measurements and pain and functional scores (VAS, HHS, and OHS). This suggests a comparable functional recovery between the two methods after 2 months postoperatively.

This study has several unique attributes. First, this study is the first to evaluate the postoperative results of the FRF method and TA for DAA. Second, the FRF method for DAA resulted in fewer surgical injuries and better clinical outcomes (VAS score, HHS, and OHS) in the first 2 months postoperatively. Third, the FRF method for DAA made the acetabular cup installation easier because of better acetabular exposure. Fourth, this study’s findings suggest that the FRF method for DAA may reduce the risk of greater trochanteric fractures and incision-related complications compared with the TA method. Fifth, the FRF method does not increase the technical requirements or operational difficulty. Finally, comparable functional recovery was observed between the FRF and TA methods for DAA after 2 months postoperatively.

This study also has several limitations. First, this was a retrospective, single-institution study with a small sample size, which is an intrinsic limitation that might introduce bias. Causality could not be confirmed in this study because of the possibility of bias. Outcomes can be affected by many factors when the techniques are used by other teams or institutions. For example, patients may not be strictly and randomly assigned to one of the two techniques; therefore, it is possible that the surgeon unwittingly selected "easier/healthier" patients to get used to a new technique. There was an increase in the use of the FRF technique during the study period, which might result in better outcomes because of the greater total experience of the surgeon. During the development of the FRF method, the study groups were small; therefore, the relevant conclusions drawn regarding the complication rate (fracture, dislocation, or infection) may be inaccurate. To further confirm the safety and effectiveness of this method, it is necessary to conduct prospective clinical studies comparing the FRF method and TA to determine which surgical procedure has the best efficacy and is associated with the least trauma. A large-sample, prospective, randomised, double-anonymised, controlled, multicentre clinical trial of DAA during THA is necessary. Second, the procedure was performed at different times, with a statistically significant follow-up duration. The experience of the surgeon and the team may have partially affected the outcomes in both groups. The learning curve was completed; however, the experience increases as the volume of surgery increases, and the operative time and surgical trauma may decrease. Third, this study had a short follow-up period, with good outcomes in the FRF group. The prosthesis survival rate and hip function outcomes at the 5-, 10-, 15-, and 20-year follow-up periods and beyond are unknown, and further observations are necessary. Finally, this study only evaluated the results for prostheses with high-edge liners. The selection range of the prosthesis was small, and the results were not compared with those for prostheses without high-edge liners. We intend to increase the number of acetabular and femoral prostheses used in future studies.

## Conclusions

Compared with the TA, employing the FRF strategy during THA with DAA resulted in improved outcomes within the first 2 months postoperatively and comparable functional recovery beyond this period. The FRF method also exhibited advantages such as favourable acetabular exposure and alignment. Therefore, the FRF strategy may be a favourable option.

## Data Availability

The datasets generated and/or analyzed during the current study are not publicly because we will enlarge the sample size and extend the follow-up time to further explore the relationship between femoral-release-first technique and clinical outcome, but these are available from the corresponding author on reasonable request through pengjiachen@139.com.

## Abbreviations

THA: Total hip arthroplasty
DAA: Direct anterior approach
TA: Traditional approach
FRF: Femoral-release-first
OA: Osteoarthritis
TFL: Tensor fasciae latae
Hb: Haemoglobin
BMI: Body mass index
ASA: American Society of Anesthesiologists
HHS: Harris Hip Score
OHS: Oxford Hip Score
VAS: Visual analogue scale
ICC: Interclass correlation coefficient
VTE: Venous thromboembolism
ERAS: Enhanced Recovery After Surgery

## References

[1] Miyamoto S, Iida S, Suzuki C, Kawamoto T, Shinada Y, Ohtori S (2022). Minimum 10-year follow-up of total hip arthroplasty with a collarless triple-tapered polished cemented stem with line-to-line implantation using a direct anterior approach. J Arthroplasty.

[2] Aggarwal VK, Elbuluk A, Dundon J, Herrero C, Hernandez C, Vigdorchik JM (2019). Surgical approach significantly affects the complication rates associated with total hip arthroplasty. Bone Joint J.

[3] Miller LE, Gondusky JS, Kamath AF, Boettner F, Wright J, Bhattacharyya S (2018). Influence of surgical approach on complication risk in primary total hip arthroplasty. Acta Orthop.

[4] Skowronek P, Wojciechowski A, Wypniewski K, Sibinski M, Polguj M, Maksymiuk-Klos A (2021). Time efficiency of direct anterior hip arthroplasty compared to postero-lateral approach in elderly patients. Arch Med Sci.

[5] Moerenhout K, Derome P, Laflamme GY, Leduc S, Gaspard HS, Benoit B (2020). Direct anterior versus posterior approach for total hip arthroplasty: a multicentre, prospective, randomized clinical trial. Can J Surg.

[6] Spina M, Luppi V, Chiappi J, Bagnis F, Balsano M (2021). Direct anterior approach versus direct lateral approach in total hip arthroplasty and bipolar hemiarthroplasty for femoral neck fractures: a retrospective comparative study. Aging Clin Exp Res.

[7] Rueckl K, Springer B, Jungwirth-Weinberger A, Bechler U, Kasparek MF, Boettner F (2021). A standardized soft tissue release technique to lower the risk of greater trochanteric fractures for the anterior approach in total hip arthroplasty. Arch Orthop Trauma Surg.

[8] Chughtai M, Samuel LT, Acuna AJ, Kamath AF (2019). Algorithmic soft tissue femoral release in anterior approach total hip arthroplasty. Arthroplast Today.

[9] Greenstein AS, Soles G (2020). Direct anterior approach to total hip arthroplasty for femoral neck fractures. J Orthop Trauma.

[10] Holzapfel BM, Rak D, Kreuzer S, Arnholdt J, Thaler M, Rudert M (2021). Short stem hip arthroplasty via the minimally invasive direct anterior approach. Oper Orthop Traumatol.

[11] Liu H, Yin L, Li J, Liu S, Tao Q, Xu J (2022). Minimally invasive anterolateral approach versus direct anterior approach total hip arthroplasty in the supine position: a prospective study based on early postoperative outcomes. J Orthop Surg Res.

[12] Hinterwimmer S, Sauerschnig M, von Eisenhart-Rothe R (2009). Diagnosis and treatment of osteoarthritis. MMW Fortschr Med..

[13] de Steiger RN, Lorimer M, Solomon M (2015). What is the learning curve for the anterior approach for total hip arthroplasty?. Clin Orthop Relat Res.

[14] Post ZD, Orozco F, Diaz-Ledezma C, Hozack WJ, Ong A (2014). Direct anterior approach for total hip arthroplasty: indications, technique, and results. J Am Acad Orthop Surg.

[15] Seagrave KG, Troelsen A, Malchau H, Husted H, Gromov K (2017). Acetabular cup position and risk of dislocation in primary total hip arthroplasty. Acta Orthop.

[16] Clement ND, Patrick-Patel RS, MacDonald D, Breusch SJ (2016). Total hip replacement: increasing femoral offset improves functional outcome. Arch Orthop Trauma Surg..

[17] Zheng W, Li J, Zhao J, Liu D, Xu W (2014). Development of a valid simplified Chinese version of the Oxford Hip Score in patients with hip osteoarthritis. Clin Orthop Relat Res.

[18] Hawker GA, Mian S, Kendzerska T, French M (2011). Measures of adult pain: visual analog scale for pain (VAS Pain), numeric rating scale for pain (NRS pain), McGill pain questionnaire (MPQ), short-form mcgill pain questionnaire (SF-MPQ), chronic pain grade scale (CPGS), short form-36 bodily pain scale (SF-36 BPS), and measure of intermittent and constant osteoarthritis pain (ICOAP). Arthritis Care Res (Hoboken).

[19] Kutzner KP, Kovacevic MP, Freitag T, Fuchs A, Reichel H, Bieger R (2016). Influence of patient-related characteristics on early migration in calcar-guided short-stem total hip arthroplasty: a 2-year migration analysis using EBRA-FCA. J Orthop Surg Res.

[20] Wroblewski BM (1991). Clinical and radiographic evaluation of total hip replacement. A standard system of terminology for reporting results. J Bone Joint Surg Am..

[21] Lewinnek GE, Lewis JL, Tarr R, Compere CL, Zimmerman JR (1978). Dislocations after total hip-replacement arthroplasties. J Bone Joint Surg Am.

[22] Wang Q, Yue Y, Yang Z, Chen L, Li Q, Kang P (2021). Comparison of postoperative outcomes between traditional longitudinal incision and bikini incision in total hip arthroplasty via direct anterior approach: a randomized controlled trial. J Arthroplasty.

[23] Gruen TA, McNeice GM, Amstutz HC. "Modes of failure" of cemented stem-type femoral components: a radiographic analysis of loosening. Clin Orthop Relat Res. 1979:17–27. 477100

[24] Callaghan JJ, Dysart SH, Savory CG (1988). The uncemented porous-coated anatomic total hip prosthesis Two-year results of a prospective consecutive series. J Bone Joint Surg Am..

[25] DeLee JG, Charnley J. Radiological demarcation of cemented sockets in total hip replacement. Clin Orthop Relat Res. 1976:20–32. 991504

[26] Engh CA, Massin P, Suthers KE (1990). Roentgenographic assessment of the biologic fixation of porous-surfaced femoral components. Clin Orthop Relat Res.

[27] Montalti M, Castagnini F, Giardina F, Tassinari E, Biondi F, Toni A (2018). Cementless total hip arthroplasty in crowe III and IV dysplasia: high hip center and modular necks. J Arthroplasty.

[28] Dihlmann W, Dihlmann SW, Hering L (1991). Alloarthroplasty of the hip joint. Radiologic diagnosis of loosening and infection in cemented total endoprostheses. Radiologe.

[29] Melloh M, Eggli S, Busato A, Roder C (2011). Predictors of early stem loosening after total hip arthroplasty: a case-control study. J Orthop Surg (Hong Kong).

[30] Landis JR, Koch GG (1977). The measurement of observer agreement for categorical data. Biometrics.

[31] Ellen S. Slovin’s Formula Sampling Techniques. sciencing.com. https://sciencing.com/slovins-formula-sampling-techniques-5475547.html. Accessed December 5.

[32] Li G, Chen Q, Zhou W, Li P, Ma P, Liu T (2022). Randomized clinical study on the efficacy of direct anterior approach combined with tendon release and repair after total hip arthroplasty. Front Surg.

[33] Dimitriou D, Helmy N, Hasler J, Flury A, Finsterwald M, Antoniadis A (2019). The role of total hip arthroplasty through the direct anterior approach in femoral neck fracture and factors affecting the outcome. J Arthroplasty.

[34] Nowak LL, Schemitsch EH (2019). Duration of surgery affects the risk of complications following total hip arthroplasty. Bone Joint J..

[35] Gruenwald KJ, Arata MA, Fisher SE (2020). Complication rates for the anterior approach to total hip arthroplasty. Orthopedics.

[36] Klasan A, Neri T, Oberkircher L, Malcherczyk D, Heyse TJ, Bliemel C (2019). Complications after direct anterior versus Watson-Jones approach in total hip arthroplasty: results from a matched pair analysis on 1408 patients. BMC Musculoskelet Disord.

[37] Spece H, Ouellette ES, Jones OL, MacDonald DW, Piuzzi NS, Lee GC (2021). Fretting corrosion, third-body polyethylene damage, and cup positioning in primary vs revision dual mobility total hip arthroplasty. J Arthroplasty.

[38] Ektas N, Scholes C, Ruiz AM, Ireland J (2020). Validity of intraoperative imageless navigation (Naviswiss) for component positioning accuracy in primary total hip arthroplasty: protocol for a prospective observational cohort study in a single-surgeon practice. BMJ Open.

[39] Knoth C, Zettl R, Markle A, Dullenkopf A, Bruhin V, Hess F (2020). A retrospective analysis of surgical outcomes following direct anterior hip arthroplasty with or without a surgical extension table. Int Orthop.

[40] Kjellberg J, Kehlet H (2016). A nationwide analysis of socioeconomic outcomes after hip and knee replacement. Dan Med J.

[41] Winemaker M, Petruccelli D, Kabali C, de Beer J (2015). Not all total joint replacement patients are created equal: preoperative factors and length of stay in hospital. Can J Surg.

[42] de Carvalho Almeida RF, Serra HO, de Oliveira LP (2021). Fast-track versus conventional surgery in relation to time of hospital discharge following total hip arthroplasty: a single-center prospective study. J Orthop Surg Res.

[43] Hansen TB (2017). Fast track in hip arthroplasty. EFORT Open Rev.

[44] Ripolles-Melchor J, Abad-Motos A, Diez-Remesal Y, Aseguinolaza-Pagola M, Padin-Barreiro L, Sanchez-Martin R (2020). Association between use of enhanced recovery after surgery protocol and postoperative complications in total hip and knee arthroplasty in the postoperative outcomes within enhanced recovery after surgery protocol in elective total hip and knee arthroplasty study (POWER2). JAMA Surg.

[45] Kawarai Y, Iida S, Nakamura J, Shinada Y, Suzuki C, Ohtori S (2017). Does the surgical approach influence the implant alignment in total hip arthroplasty? Comparative study between the direct anterior and the anterolateral approaches in the supine position. Int Orthop.

[46] Luger M, Hochgatterer R, Klotz MC, Allerstorfer J, Gotterbarm T, Schauer B (2022). A single-surgeon experience in reconstruction of femoro-acetabular offset and implant positioning in direct anterior approach and anterolateral MIS approach with a curved short stem. Arch Orthop Trauma Surg.

